# The association of five preoperative serum tumor markers and pathological features in patients with breast cancer

**DOI:** 10.1002/jcla.22875

**Published:** 2019-03-06

**Authors:** Mingjian Lian, Cuixia Zhang, Dongdong Zhang, Ping Chen, Huijing Yang, Yuanyuan Yang, Shidong Chen, Guolin Hong

**Affiliations:** ^1^ Department of Clinical Laboratory The First Affiliated Hospital of Xiamen University Xiamen China; ^2^ Department of Pathology The First Affiliated Hospital of Xiamen University Xiamen China; ^3^ Medical Department Fujian Medical University Fuzhou China

**Keywords:** breast cancer, molecular subtype, pathological feature, prognosis, tumor marker

## Abstract

**Background:**

The utility of frequently used serum tumor markers in breast cancer remains controversial. The study aimed to investigate the role of preoperative carcinoembryonic antigen (CEA), cancer antigen 125 (CA125), cancer antigen 153 (CA153), cancer antigen 724 (CA724), and ferritin (FER) in the management of breast cancer and their relationships with pathological features.

**Methods:**

A total of 804 patients with breast mass who underwent breast surgery and 305 healthy volunteers were enrolled. Preoperative serum levels of CEA, CA125, CA153, CA724, and FER were measured. And the pathological features of all the patients were recorded. The association of preoperative serum tumor markers and pathological features was analyzed.

**Results:**

Among the 804 patients, 355 were identified as malignant cases and 449 as benign cases. CEA, CA153, and FER of patients with breast cancer were higher than those of healthy volunteer group and patients with benign breast diseases. The area under curve (AUC) of CEA, CA153, and FER for distinguishing patients with breast cancer and subjects with non‐breast cancer was 0.688 (95% CI: 0.656‐0.721), 0.609 (95% CI: 0.574‐0.645), and 0.623 (95% CI: 0.586‐0.660), respectively. CA153 correlated with tumor size, node status, and TNM stage, whereas CA125 with node status. No statistic differences of the five markers were observed among the four molecular subtypes.

**Conclusion:**

Preoperative levels of CEA, CA153, and FER exhibit low diagnostic accuracy for breast cancer (stage I‐III). CA153 correlates with tumor burden, suggesting its prognostic value. The five serum markers do not correlate with molecular subtypes.

## 1 INTRODUCTION

Breast cancer is the most frequent malignancy in women, and its incidence has been steadily increasing these years. It was estimated that 272 400 breast cancer cases were newly diagnosed in China in 2015, corresponding to almost 70 700 breast cancer deaths.^1^ The survival of breast cancer is significantly dependent on the stage of cancer at diagnosis. Currently, the diagnosis of breast cancer is mainly dependent on imaging examinations and/or pathological results. And the treatment and prognosis are closely related to pathological features, such as pathological type, tumor size, node status, TNM stage, hormone receptor status, and molecular subtype. According to the expression of estrogen receptor (ER), progesterone receptor (PR), human epidermal growth factor receptor 2 (HER‐2), and Ki‐67, breast cancer is classified into four molecular subtypes, including Luminal A, Luminal B, HER‐2‐positive, and triple‐negative.^2^ As reported, Luminal A showed the best survival among the four subtypes, while triple‐negative breast cancer showed the worst.^3^, ^4^ Therefore, molecular subtypes are important for individual treatment and have different prognostic values.

Serum markers can be easily achieved, and their clinical values have been investigated in breast cancer. Cancer antigen 153 (CA153) and carcinoembryonic antigen (CEA) are the most commonly used serum markers in the management of breast cancer. A meta‐analysis including 12 993 patients pointed out that elevated serum CA153 or CEA was associated with poor disease‐free survival and overall survival in breast cancer.^5^ In recent years, a number of studies have focused on the association of serum tumor markers and clinicopathological features, but inconsistent results were reported. Wu et al found that patients with triple‐negative breast cancer had the lowest CEA level among the four subtypes, whereas Fang et al found elevated cancer antigen 125 (CA125) was more frequently observed in triple‐negative patients compared with other three subtypes and they did not find any difference of serum CEA and CA153 among the four subtypes.^6^, ^7^ Thus, the limited knowledge of the relationship between serum tumor markers and the pathological features has obstructed the optimized use of serum tumor markers for patients with breast cancer.

Herein, we performed a retrospective study to compare preoperative serum levels of five tumor markers, including CEA, CA125, CA153, cancer antigen 724 (CA724), and ferritin (FER), among patients with breast cancer, patients with benign breast diseases, and healthy volunteers. Additionally, the diagnostic accuracy of the tumor markers for breast cancer was evaluated, and the association of preoperative serum markers with pathological features was analyzed to provide more evidence for clinical practice.

## 2 MATERIALS AND METHODS

### 2.1 Study subjects

A total of 804 patients with breast mass who had breast surgery were enrolled consecutively between July 2016 and June 2017 in the First Affiliated Hospital of Xiamen University. The inclusion criteria were (a) with no history of cancer; (b) with complete medical record; (c) serum tumor markers were detected within two weeks prior to surgery; and (d) without radiotherapy/chemotherapy/endocrinotherapy before surgery. We excluded patients with unknown TNM stage, male breast cancers, secondary cancer, and patients with stage IV disease at diagnosis. During these periods, 305 age‐matched healthy volunteers were enrolled as healthy volunteer group. Written informed consents were obtained from all subjects included in the study for the use of their medical records for research purposes, and the study design and method were approved by the Ethics Committee of the First Affiliated Hospital of Xiamen University.

### 2.2 Serum tumor markers and pathological features

Three milliliters of venous blood was collected from all patients before surgery. After centrifugation, serum CEA, CA125, CA153, and FER were measured using automatic chemiluminescence immunoassay system (SIEMENS ADVIA centaur; Siemens, Germany), and serum CA724 was measured using ROCHE E601 (Roche, Germany). A cutoff limit of 5 ng/mL (CEA), 35 U/mL (CA125), 32.4 U/mL (CA15‐3), 8.2 U/mL (CA724), and 291.0 µg/L (FER) was used as recommended by the manufacturer.

Immunohistochemistry (IHC) method was used to detect the expression of ER, PR, HER‐2, and Ki‐67. ER‐positive and PR‐positive were defined as the presence of 1% nuclear‐stained cells. HER2‐positive was indicated by a 3+ score from the immunohistochemical evaluation. The Ki‐67 staining was considered to be positive if the percentage was >14%. The breast tumor was classified into four subtypes in accordance with the St. Gallen International Expert Consensus.^2^ And the TNM stage was evaluated by American Joint Committee on cancer (AJCC) staging system.^8^

### 2.3 Statistical analysis

All data were analyzed using SPSS 17.0 software (SPSS; Chicago, IL). The Mann‐Whitney test was applied to analyze the differences between quantitative data for two independent groups and the Kruskal‐Wallis test for three independent groups. Receiver operating characteristic curve (ROC) analysis was used to evaluate the diagnostic accuracy. If CEA was <0.5, 0.5 ng/mL was used for analysis instead. *P < *0.05 was considered to be statistically significant.

## 3 RESULTS

### 3.1 Patient characteristics

According to the inclusion and exclusion, 804 patients with breast mass who underwent breast surgery were eventually enrolled, including 355 breast cancer cases and 449 benign cases. And 305 healthy volunteers were enrolled as healthy volunteer group. The median age was 44 years (range, 11‐81 years) and 45 years (range, 21‐80 years) in patients with breast mass and healthy volunteers, respectively. Invasive ductal carcinoma (IDC) was the most common pathological type, accounting for 80.3% of cancer patients. Out of the 355 cancer patients, 40 patients (11.3%) were classified as Luminal A, 245 patients (69.0%) as Luminal B, 32 patients (9.0%) as HER‐2‐positive, and 38 patients (10.7%) as triple‐negative. Among the 355 cancer patients, TNM stage I, stage II, and stage III accounted for 34.1%, 44.8%, and 21.1%, respectively. And a majority of benign patients were patients suffering with mammary hyperplasia or fibroadenoma.

### 3.2 Preoperative serum levels of tumor markers

The preoperative serum levels of tumor markers in different groups are presented in Table 1. CEA, CA153, and FER of patients with breast cancer were higher than those of healthy volunteer group and patients with benign breast diseases (*P* < 0.05). In comparison with the healthy volunteer group, both patients with breast cancer and patients with benign breast diseases had higher CA125 (*P* < 0.05). However, there was no significant difference of CA724 among the three groups. In addition, the positive rates of tumor markers in three groups were calculated (as shown in Table 2).

**Table 1:** Preoperative tumor markers levels of the study population (n = 1109)

Tumor markers	HV group (n = 305)	Benign (n = 449)	Malignant (n = 355)
	Median (P25, P75)	Median (P25, P75)	Median (P25, P75)
CEA (ng/mL)	0.59 (0.05, 1.11)	0.61 (0.50, 1.16)	1.17 (0.56, 1.80)*,**
CA125 (U/mL)	8.75 (6.50, 12.02)	11.90 (8.80, 15.95)*	11.00 (8.10, 15.60)*
CA153 (U/mL)	5.60 (3.60, 8.85)	5.40 (3.40, 8.70)	6.90 (4.70, 11.40)*,**
CA724 (U/mL)	1.84 (1.17, 3.20)	1.66 (0.96, 4.45)	1.64 (0.89, 3.63)
FER (µg/L)	45.50 (22.40, 90.85)	48.40 (25.15, 93.30)	81.10 (35.00,157.10)*,**

CA125, cancer antigen 125; CA153, cancer antigen 153; CA724, cancer antigen 724; CEA, carcinoembryonic antigen; FER, ferritin; HV group, healthy volunteer group.

* Compared with HV group, *P* < 0.05.

** Compared with Benign, *P* < 0.05.

**Table 2:** Positive rates of tumor markers in different groups

Tumor markers	HV group (%)	Benign (%)	Malignant (%)	*P*
CEA	0.98	0.22	3.1	<0.01*
CA125	3.28	1.78	3.66	0.230
CA153	0.00	0	1.97	<0.01*
CA724	7.54	11.8	10.7	0.159
FER	2.30	2.67	9.01	<0.001*

CA125, cancer antigen 125; CA153, cancer antigen 153; CA724, cancer antigen 724; CEA, carcinoembryonic antigen; FER, ferritin.

* *P* < 0.05 indicates a significant difference.

**Figure 1:**
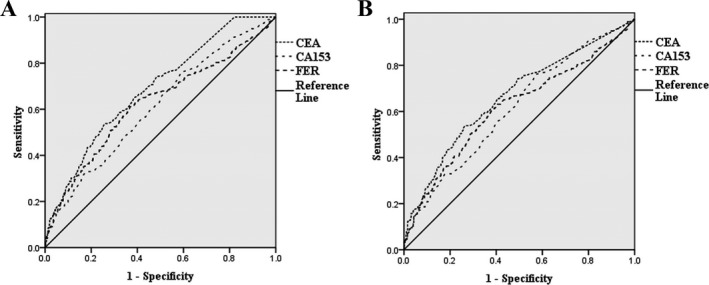
Receiver operating characteristic curve was constructed to evaluate the diagnostic accuracy of tumor markers for breast cancer. A, for distinguishing patients with breast cancer and subjects with non‐breast cancer, B, for distinguishing patients with breast cancer and patients with benign breast diseases

To evaluate the diagnostic accuracy of CEA, CA153, and FER for breast cancer, ROC analyses were performed (as shown in Figure 1). The area under curve (AUC) of CEA, CA153, and FER for distinguishing patients with breast cancer and subjects with non‐breast cancer (healthy volunteer group and patients with benign breast diseases) was 0.688 (95% CI: 0.656‐0.721), 0.609 (95% CI: 0.574‐0.645), and 0.623 (95% CI: 0.586‐0.660), respectively. And the AUC of CEA, CA153, and FER for distinguishing patients with breast cancer and patients with benign breast diseases was 0.663 (95% CI: 0.625‐0.701), 0.613 (95% CI: 0.574‐0.652), and 0.615 (95% CI: 0.575‐0.655), respectively.

### 3.3 Serum markers and TNM stage

**Figure 2:**
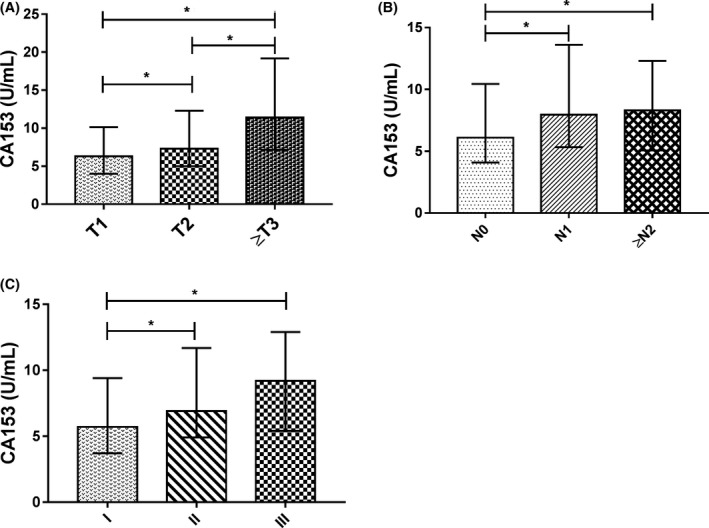
The association of preoperative serum CA153 levels with tumor size (A), node status (B), and TNM stage (C) in patients with breast cancer. *P < 0.05

As shown in Table 3 and Figure 2, the preoperative serum level of CA153 had a rise trend along with the development of tumor. Among patients with breast cancer, the level of CA153 was associated with tumor size. Patients with (≧T3) had higher CA153 level than both patients with T2 and patients with T1, and patients with T2 had higher CA153 level than patients with T1. As to node status, the levels of CA153 were significantly elevated in both patients with N1 and patients with (≧N2) when compared to patients with N0 (*P* < 0.05). Likewise, the levels of CA153 were significantly elevated in both stage II patients and stage III patients when compared to stage I patients (*P* < 0.05). Besides, a statistical difference of CA125 was found among the three groups of node status (*P* = 0.043).

**Table 3:** Relationship between serum marker levels and pathological features in patients with breast cancer

	N	CEA (ng/mL)	*P*	CA125 (U/mL)	*P*	CA153 (U/mL)	*P*	CA724 (U/mL)	*P*	FER (µg/L)		*P*
		Median (P25, P75)		Median (P25, P75)		Median (P25, P75)		Median (P25, P75)		Median (P25, P75)		
Size												
T1	166	1.09 (0.50, 1.70)	0.237	10.75 (8.10, 15.23)	0.056	6.30 (4.00, 10.13)	<0.001*	1.58 (0.89, 3.35)	0.686	75.20 (33.33, 149.25)		0.483
T2	167	1.22 (0.65, 1.88)		10.70 (7.90, 15.90)		7.30 (5.00, 12.30)		1.69 (0.89, 4.15)		83.30 (36.50, 166.00)		
≧T3	22	1.43 (0.73, 2.16)		13.75 (10.80, 21.73)		11.40 (7.18, 19.18)		1.86 (0.88, 4.25)		91.20 (51.13, 200.73)		
Node status												
N0	205	1.13 (0.56, 1.82)	0.212	10.50 (7.70, 14.70)	0.043*	6.10 (4.10, 10.45)	0.001*	1.53 (0.88, 3.50)	0.141	70.50 (35.75, 147.45)		0.396
N1	80	1.09 (0.50, 1.72)		11.80 (8.48, 14.78)		7.95 (5.33, 13.60)		2.20 (0.99, 4.50)		102.20 (37.13, 178.95)		
≧N2	70	1.37 (0.74, 1.80)		12.20 (8.80, 21.95)		8.30 (5.08, 12.30)		1.52 (0.88, 3.51)		84.25 (25.28, 156.48)		
TNM stage												
I	121	1.12 (0.53, 1.84)	0.397	9.90 (7.90, 13.30)	0.132	5.70 (3.70, 9.40)	<0.001*	1.30 (0.88, 3.04)	0.064	75.20 (34.90, 136.85)		0.811
II	159	1.14 (0.50, 1.80)		11.60 (8.00, 15.60)		6.90 (4.90, 11.70)		2.02 (0.98, 4.15)		81.50 (33.50, 172.20)		
III	75	1.35 (0.72, 1.77)		11.30 (8.80, 18.60)		9.20 (5.40, 12.90)		1.51 (0.88, 3.33)		88.30 (37.80, 157.10)		
ER												
Positive	270	1.17 (0.56, 1.79)	0.695	11.00 (8.08, 15.03)	0.474	7.10 (4.38, 11.43)	0.612	1.62 (0.89, 3.51)	0.699	80.65 (34.55, 149.53)		0.611
Negative	85	1.19 (0.60, 1.95)		10.60 (8.70, 16.90)		6.50 (4.95, 11.05)		1.70 (0.88, 5.02)		81.60 (36.25, 194.10)		
PR												
Positive	252	1.08 (0.52, 1.71)	0.031*	11.00 (8.10, 15.60)	0.991	7.10 (4.55, 11.48)	0.537	1.65 (0.89, 3.50)	0.526	73.50 (30.73, 145.33)	0.032*	
Negative	103	1.39 (0.65, 1.99)		10.80 (8.20, 15.40)		6.70 (4.80, 10.90)		1.61 (0.92, 4.46)		96.20 (40.90, 198.60)		
HER‐2												
Positive	91	1.18 (0.63, 1.86)	0.775	10.80 (8.20, 16.20)	0.896	6.00 (4.50, 10.20)	0.331	1.74 (0.95, 4.24)	0.576	68.40 (28.20, 149.40)	0.183	
Negative	264	1.17 (0.54, 1.79)		11.00 (8.10, 15.38)		7.20 (4.70, 11.50)		1.63 (0.89, 3.58)		83.50 (37.15, 172.10)		
Ki‐67												
Positive	310	1.19 (0.60, 1.80)	0.405	10.90 (8.10, 15.45)	0.836	7.20 (4.70, 11.50)	0.297	1.71 (0.88, 3.75)	1.000	81.70 (33.73, 157.03)	0.857	
Negative	45	1.02 (0.50, 1.79)		11.20 (8.45, 15.95)		6.10 (4.80, 10.75)		1.30 (0.96, 2.93)		75.20 (42.00, 166.20)		
Subtype												
Luminal A	40	1.03 (0.50, 1.75)	0.865	11.35 (8.43, 16.13)	0.444	6.25 (4.30, 11.35)	0.182	1.61 (0.97, 3.52)	0.873	75.20 (41.55, 169.00)	0.823	
Luminal B	245	1.19 (0.60, 1.80)		10.90 (8.05, 10.05)		7.40 (4.60, 11.70)		1.60 (0.88, 3.49)		82.10 (33.65, 151.75)		
HER‐2 (+)	32	1.13 (0.62, 1.92)		10.45 (6.40, 15.25)		5.35 (4.83, 7.23)		1.48 (0.90, 5.60)		76.15 (17.80, 154.63)		
Triple‐negative	38	1.24 (0.50, 1.81)		12.60 (9.30, 20.23)		7.05 (4.85, 11.48)		1.75 (0.90, 4.55)		82.45 (39.35, 199.90)		

CA125, cancer antigen 125; CA153, cancer antigen 153; CA724, cancer antigen 724; CEA, carcinoembryonic antigen; ER, estrogen receptor; FER, ferritin; HER‐2, human epidermal growth factor receptor 2; P25, the 25th percentile; P75, the 75th percentile; PR, progesterone receptor.

* *P* < 0.05 indicates a significant difference.

### 3.4 Serum markers and immunohistochemical results

As shown in Table 3, significantly elevated CEA and FER were found in PR‐negative group when compared to PR‐positive group (*P* < 0.05). There were no statistical differences of the five markers between ER‐positive group and ER‐negative group, between HER‐2‐positive group and HER‐2‐negative group, and between Ki‐67‐positive group and Ki‐67‐negative group (*P* > 0.05). In addition, preoperative serum level of CA153, CA125, CA153, CA724, and FER showed no statistical differences among the four molecular subtypes (*P* > 0.05).

## 4 DISCUSSION

Compared with the pathological examination, serum tumor markers have several advantages. To evaluate the clinical application of serum markers is of great significance. For the first time, we explored the association between preoperative levels of five serum tumor markers and pathological features of breast cancer patients, and evaluated clinical values of these five tumor markers for breast cancer.

Consistent with previous studies,^6^, ^9^, ^10^ elevated serum levels of CEA and CA153 were observed in breast cancer patients. In our study, FER was also found to be elevated in breast cancer. As known, FER, the primary iron storage protein, is increased in multiple human malignancies. Considering elevated CEA, CA153, and FER were observed in breast cancer, the diagnostic accuracy of them was analyzed. ROC analyses revealed that all of the three markers had low diagnostic accuracy (AUC < 0.700) for discriminating patients with breast cancer from subjects with non‐breast cancer as well as for discriminating patients with breast cancer from patients with benign breast diseases. As most of patients with stage IV did not receive surgical treatment and pathological features were known, patients with stage IV were excluded in our study. As reported by Wang’s group, serum CEA, CA125, and CA153 were found to be higher in metastatic breast cancer patients than breast cancer patients without metastasis.^11^ Thus, we can only conclude that CEA, CA153, and FER had low diagnostic accuracy for early‐stage breast cancer (stage I‐III). The positive rates of tumor markers in three groups were below 15%, indicating low sensitivity and high specificity of them for diagnosing breast cancer. From this point of view, the low sensitivity limited their usage for screening early stage of breast cancer and high specificity indicated that much attention should be paid to positive results.

Previous researches demonstrated that serum tumor markers such as CEA, CA125, and CA153 were associated with tumor burden indicators including tumor size, node status, and TNM stage.^6^, ^7^ In our study, only CA153 shared the similar result. Serum level of CA153 increased with the development of breast cancer, suggesting its prognostic value. As reported, serum CA153 level was associated with tumor metastasis.^12^, ^13^ In addition, Tampellini et al reported that CA153 levels were elevated in breast cancer patients with liver metastases.^14^ Thus, when it comes to high CA153, more attention should be paid. Besides, CA125 was found to be associated with axillary lymph node status in our study. However, we did not observe the association between CAE/CA724/FER and tumor burden.

It is known that ER plays a role in cellular growth, proliferation, and differentiation. Both ER and PR are tumor markers that can effectively predict the hormonal responsiveness.^15^ HER‐2 has been proposed to estimate prognosis and guide treatment. Ki‐67 is considered to be a proliferation index. Thus, molecular subtypes based on the results of ER, PR, HER‐2, and Ki‐67 are of great importance for clinicians. And to analyze the association of serum tumor markers with immunohistochemical results seemed to be meaningful. Unfortunately, we did not find any statistic differences of serum CEA, CA125, CA153, CA724, and FER among the four subtypes in the current study. In agreement with Fang's results, they found that CEA and CA153 did not correlate with molecular subtypes, but they found that CA125 exhibited statistical differences among various molecular subtypes, with the most frequent elevations occurring in the triple‐negative tumors.^7^ As for metastatic breast cancer, Geng et al^16^ concluded that elevated CA 153 and CEA levels at initial diagnosis of recurrence were found to be associated with breast cancer molecular subtypes. In addition, Wu^6^ and his colleagues reported that CEA levels were lower in patients with triple‐negative breast cancer than patients with other subtypes, and CA153 did not correlate with molecular subtypes. Thus, the association of serum tumor markers and molecular subtypes is not conclusive, and further studies with larger sample are needed. Additionally, our results showed both CEA and FER levels were greater in PR‐negative group than in PR‐positive group. Consistent with Imamura's results, they also found the elevated CEA was more frequent in PR‐negative group than PR‐positive group.^17^

There are some limitations should be acknowledged. First, as a retrospective study, the available data may have some biases. Second, the level of tumor markers may be affected by several factors, such as age, region, body mass index, lifestyle, and environment. Third, small sample size should be acknowledged. Nevertheless, this study contains a number of strengths. First, the inclusion and exclusion criteria were rigorous. Second, for the first time, five serum tumor markers prior to surgery were analyzed. Third, in despite of negative result, the study may be helpful in better understanding the association of the five serum markers with molecular subtypes.

In summary, our study indicated that preoperative serum levels of CEA, CA153, and FER were elevated in breast cancer patients, with low diagnostic accuracy for breast cancer (stage I‐III). High preoperative CA153 may reflect tumor burden, suggesting that it can be used to predict prognosis of breast cancer patients and monitor advanced tumors. Additionally, there was no significant association of preoperative tumor marker levels with molecular subtypes. Therefore, further studies are needed to find out better tumor markers for the diagnosis and prognosis of breast cancer.

## CONFLICT OF INTEREST

None.
